# Supplementary material for: Kinetics and mechanical work done to move the body centre of mass along a curve
Source: PLoS One. 2024 Feb 12;19(2):e0298790. doi: 10.1371/journal.pone.0298790 (PMC10861085; doi:10.1371/journal.pone.0298790)
Supplement: S1 Fig — (DOCX) [file pone.0298790.s001.docx]

**Fig S1** The rotated oscillation of *PL_c_* (in red) over two strides and the rotated displacement of the CoM (in black) calculated as the double integration of the horizontal GRF using the initial and final positions of the *PL_c_* and the duration of the trial (*T*_tot_) as the integration constant for (*p_x_, p_y_*)*.* The trace is run at 14 km h^-1^ and on a 6 m radius of curvature and is from the same trial as in Fig 1B. The scales are asymmetrical to accentuate the oscillations around the curve.
