# Supplementary material for: Kinetics and mechanical work done to move the body centre of mass along a curve
Source: PLoS One. 2024 Feb 12;19(2):e0298790. doi: 10.1371/journal.pone.0298790 (PMC10861085; doi:10.1371/journal.pone.0298790)
Supplement: S2 Fig — (DOCX) [file pone.0298790.s002.docx]

A

B

**Fig S2** (A) The typical energy traces (J) of one stride in the o-*x-y-z* referential from the first right stride used in Figure 1 at both radii plus the typical trace of one stride from when the subject ran on a treadmill at the same speed (14 km h^-1^). The blue represents the outer leg and red the inner. *E*_z_ is the energy done to sustain the vertical movements of the CoM. *E*_x_ is the energy done to sustain the medio-lateral movements of the CoM and *E_y_,* similarly but for the fore-aft movements. *E*_com_ is the sum of all three first curves. (B) The time-normalised rotated GRFs (*F*_Y_ and *F*_X_, expressed in Newtons) for every inner (red) and outer step (blue) done at 14 km ^-1^ for the 18 m and 6 m radii based on the continuously rotated referential using the θ- angle as explained below. The time-normalised typical energy traces (J) of $E_{X}$ and $E_{Y}$ for all the steps at the same speed calculated similarly from *F*_X_, *F*_Y_ forces above.

### **Classic method:** Computation of external energy relative to a fixed referential frame

When running along a circular path, the *y*-direction no longer corresponds to the fore-aft direction of progression. Let’s take, for example, a subject that runs on a 90° circular curve turning to the right. If the external referential is fixed to the lab, the subject runs in the *y-*direction at the beginning of the curve, and in the *x-* direction at the end of the curve. Thus, the CoM progressively gains velocity along the *x* axis and loses velocity along the *y*-axis. In other words, during the movement, the kinetic energy *E*_y_ (equation 11) is decreasing while the kinetic energy *E*_x_ (equation 10) is increasing (Fig. S2A) The energy of the CoM due to its vertical movement (*E*_z_) is not affected by the turn.

In this case, the total work *W*_com_ is correctly measured, however the division of the work into *W*_x_ and *W*_y_ has no physiological meaning, and the distinction between the work done to sustain the motion of the CoM in the direction of progression (*W*_f_) and in the lateral direction (*W*_l_, *i.e.,* in the direction perpendicular of the axis of progression) is not possible.

***Continuous rotation of the forces:*** *Computation of the external energy relative to a referential frame rotating at each instant of the step*

In this second approach one a more obvious solution for rotating the referential should be to rotate the frame of reference at each instant so that the *Y*-axis of the new reference frame is tangent to the circular trajectory that the runner follows, and that the *X*-axis is pointed towards the centre of the circle. However, in this case, it is difficult to separate the work done to move the CoM in the axis of progression (*W*_f_) from the work necessary to modify the direction of progression (*W*_l_). The *θ* -angle is used for this rotation:

$\theta= 180^{\circ}-\mathrm{atan}\frac{{PLC}_{cy}-y_{o}}{{PLC}_{cx}-x_{o}}$ .

The two horizontal components of the GRF in the frame *O-X-Y* were computed as:

$\left( F_{X} F_{Y} \right)=\left( \begin{matrix} \cos\theta& -\sin\theta\\ \sin\theta& \cos\theta\end{matrix} \right) \left( \begin{matrix} F_{x} \\ F_{y} \end{matrix} \right)$ .

Similarly, the two horizontal components of the velocity of the CoM were computed as:

$\left( v_{X} v_{Y} \right)=\left( \begin{matrix} \cos\theta& -\sin\theta\\ \sin\theta& \cos\theta\end{matrix} \right) \left( \begin{matrix} v_{x} \\ v_{y} \end{matrix} \right)$ .

Then the energy of the CoM due to the velocity changes in the *Y*-axis, and the *X*-axis were computed using equations similar to equations (6) and (7) in the new reference frame:

$E_{Y}=\int F_{Y} v_{Y}dt = \frac{1}{2} m {v_{\text{Y}}}^{2}$ ,

$E_{X}=\int F_{X} v_{X}dt= \frac{1}{2} m {v_{\text{X}}}^{2}$,

Note that because we are using *R* and not *r* for the calculation of θ, there is a slight amount of energy done in the centripetal axis. If one were to consider the centripetal axis at each instant, no energy is done as the force and velocity vectors are orthogonal. Nevertheless, nearly all of the energy done in the horizontal plane is done in the direction tangent to the circle. Second, the energy of the CoM due to its vertical movement (*E*_z_) is not affected by the turn. The total energy of the CoM (*E*_com_) is then computed by:

${E_{\text{com}}=E}_{\text{Y}}+ E_{\text{X}}{+ E}_{\text{z}}$ .
