# Supplementary material for: Kinetics and mechanical work done to move the body centre of mass along a curve
Source: PLoS One. 2024 Feb 12;19(2):e0298790. doi: 10.1371/journal.pone.0298790 (PMC10861085; doi:10.1371/journal.pone.0298790)
Supplement: S3 Fig — (DOCX) [file pone.0298790.s003.docx]

**Fig S3** The average difference between the ‘instantaneous’ radius, *r*, and the actual radius *R* (*r-R*) calculated by the circular fit from the movements of *PL*_c_ as explained in the methods, plotted as a function of time. The top panels show three different speeds (10, 14 and 17 km h^-1^) when running on an 18 m curve, whereas below the same speeds are shown for a 6 m curve. The average trace (thick line) is calculated over all participants and the cloud around is the standard deviation. Time is expressed in a percentage of the stride period. The traces start on the inner leg *TD* where the distance between *r* and *R* is at its highest (in red), before the CoM trajectory approaches the circle and the distance between both is reduced prior to the aerial phase (green trace). This is followed by the outer step (blue trace) which has a similar negative distance to *R* than the inner leg and is also progressively directed inwards prior to the second aerial phase. Note, during the aerial phase the CoM is travelling in a rectilinear motion. Here above, the aerial phase curves because we are looking at a difference between the CoM trajectory and the curve. Therefore, if the CoM begins its flight close to the circle and continues straight whereas the curve bends then the distance between both increases.
