# Supplementary material for: Kinetics and mechanical work done to move the body centre of mass along a curve
Source: PLoS One. 2024 Feb 12;19(2):e0298790. doi: 10.1371/journal.pone.0298790 (PMC10861085; doi:10.1371/journal.pone.0298790)
Supplement: S4 Fig — (DOCX) [file pone.0298790.s004.docx]

**Fig S4** The variation of foot angle ($\tau$) in the horizontal plane while the foot was flat on the ground as a function of speed at both radii of curvature. $\tau$ was defined as the angle formed between the *x-y* position of the markers placed on the 5^th^ metatarsal, the heel and the horizontal. The variation was measured as the standard deviation of $\tau$ between the point when the heel touches the ground until when the heel lifted off the ground by 0.05m, or so when the foot was flat on the ground. This was chosen as a means to verify that the foot was not skidding or rotating over the ground rather than measuring the ankle-roll over the toes during contact. All other indications are as in Fig 4.
